# Medicine as She Is Taught

**Published:** 1894-08

**Authors:** 


### MEDICINE AS SHE IS TAUGHT.

During the last meeting of the legislature the able counsel for the notorious quack and fraud, Dr. Flower, of Boston, stated before the Committee of the House that he doubted the existence of the so-called quack medical colleges. We have looked up some of these, where diplomas may be obtained—for a moderate consideration.

There are, or have been, about twenty of these diploma mills in the United States. The examining boards have them all on the list.

A "Physio-Medical College" is located at Indianapolis, of which "Professor" Josiah Adams seems to be the chief official. In a late letter to a medical student "Professor" Adams wrote: "Wea Will Give you Beter terms And Beter instructions than you Can Get iny other plase in the united States, etc. * * * * You Will Bea Graduated at iny time on A persint of Sixty-five." The Professor's College is "on the list."

Cincinnati has more than her share of these miserable concerns. There is the "Medical University of Ohio," of which one, Van Vleck, an itinerant quack, is President. Not long ago this Van Vleck served a term in jail for some of his rascality. "Nature's Healing College" grinds out doctors after a six weeks' course. The "Vita-Pathic Institute," and the "American Eclectic Institute" also sell diplomas on short notice. There is also a Christian science institution, and another of the Faith Cure persuasion, all of which seem to be doing a thriving business.* These are "on the list."

Out in Missouri there is a medical college, so-called, consisting of a three-story brick building owned by a Dr. French. "On the ground floor in the front the Doctor has his private office; in the rear of this office, on the same floor, is his family residence. The second floor is devoted to the purpose of a private hospital. On the third floor is the medical college. There is one poorly furnished lecture-room, and the equipments consist of a few cheap charts hung upon the walls, a blackboard, and an articulated skeleton, the bones appearing as if they

* Cincinnati *Lancet-Clinic.*

had been exhumed several years after death. Apparatus for teaching chemistry, bacteriology, and physiology, there is none. The dissecting-room, which is on the third floor, is very clean, for the reason that there has never been a cadaver in it."* This College is "on the list."

There are several of these concerns in Chicago, the "Chicago Correspondence University," the "National University of Illinois," the "German Academy of Physiatric Physicians," etc. These are also "on the list." Out in Washington there is the "Northwestern College of Biochemistry," which was inaugurated some time ago by a certain G. W. Carey. He organized the "College," made himself Dean and President, then conferred upon himself the title, "M.D.," and proceeded to dispense diplomas. This great "biochemist" and his college are also "on the list."

These are not all, but they are enough to point a moral—the necessity for some legislation which will protect the people from such shameless institutions and their graduates. The weather eyes of our State Boards are on them. They are all "on the list." They stand convicted of fraud and are doomed to go. "They never will be missed, they never will be missed."

